# Morphological abnormalities in *Afrixodes* ticks

**DOI:** 10.1016/j.ijppaw.2026.101258

**Published:** 2026-07-01

**Authors:** Camille Lorang, Denis Augot

**Affiliations:** a ANSES, INRAE, Ecole Nationale Vétérinaire d’Alfort, UMR BIPAR, Laboratoire de Santé Animale, Maisons-Alfort, 94700, France; b Oniris, INRAE, BIOEPAR, Nantes, France

**Keywords:** Teratology, *Ixodes*, Wildlife

## Abstract

In mythology, teratology refers to fantastic creatures and monsters, but the “science of monsters” in biology refers to congenital abnormalities and abnormal formations. These anomalies are well reported in ticks, but only a few of them deal with *Ixodes* ticks, ticks collected from wildlife, or ticks from Africa. This study investigated a large collection of Afrotropical ticks, *Ixodes* (*Afrixodes*), collected from wildlife, which included two nymphs (*Ixodes* (*A*.) spp.) and one adult female (*I. rasus*) that turned out to be teratological specimens, presenting ectromely (missing leg). The present work represents the first report of morphological abnormalities in *Ixodes (A.) rasus* and *Ixodes* (*A.*) spp. collected on Muridae, Soricidae and Tragulidae in the Central African Republic and Gabon.

## Highlights

• Two nymphs (*Ixodes* (*A*.) sp.), one female (*Ixodes* (*A*.) *rasus*) present ectromely.
• This represents 0.45% of ticks from the collection that have distortion.
• First report for ticks collected on Muridae, Soricidae, Tragulidae, in Central African Republic and Gabon.

## Graphical abstract

**Figure:**
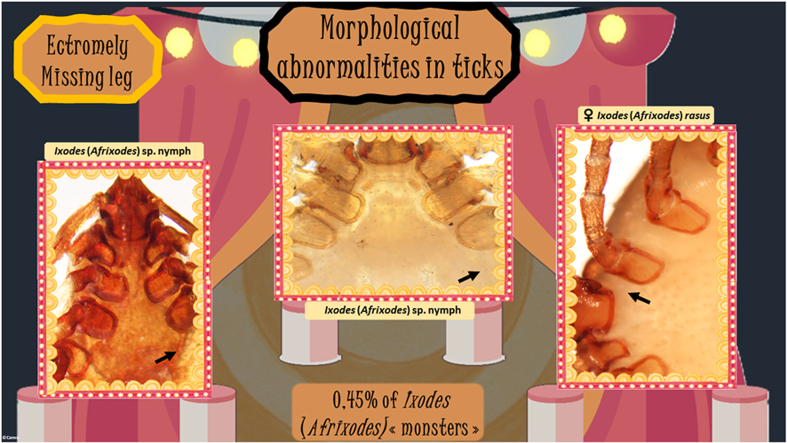

## 1 Introduction

Morphological abnormalities have been described since the 18th century for both insects and ticks by Neumann (1899). Teratology – the science of “monsters” – aims to describe these abnormalities, which are divided into general abnormalities (gynandromorphism, double deformity, dwarfism, etc.) and local ones, as shown by Campana-Rouget in 1959 (Campana-Rouget, 1959a). This is especially applicable to ticks. Local abnormalities include the atrophy of certain appendices, the presence of an additional leg or appendices (polymely), different kind of leg fusion (heterosymely, symely) or the absence of a leg (ectromely) or a pedipalp (oligomely) (Campana-Rouget, 1959b). These morphological abnormalities can be due to several biotic or abiotic causes, and often prove difficult to identify. Teratological abnormalities could be caused by an injury in the stage before the one when the abnormality is found, common for ectromely (Campana-Rouget, 1959a; Chitimia-Dobler et al., 2017), chemical pollution of the tick's environment (Buczek et al., 2013), temperature (Buczek, 2000) or a change in the usual host (Nowak-Chmura, 2012). So, it is difficult to determine the exact cause of these abnormalities. Buczek (2000) has nevertheless demonstrated experimentally that temperature can affect embryogenesis and, among other things, lead to abnormal development in larvae, including ectromely.

Campana-Rouget (1959a) revealed the rarity of these abnormalities through literature that has remained relevant to this day (Bykov et al., 2023; Chitimia-Dobler et al., 2017). Teratological references to ticks found on wild fauna (in Türkiye by Keskin, 2018; in Brazil by Luz et al., 2023), or in their nest (from birds *Riparia riparia* in Iran by Bykov et al., 2023) or domestic animals (Keskin, 2018; Balinandi et al., 2019; Shuaib et al., 2020) are rarer than those found during flagging studies in vegetation (Kar et al., 2015; Larson and Paskewitz, 2016). Teratological ticks have also been reported from lab experiments (Buczek et al., 2013; Taank et al., 2021). Most of these studies focused on Palearctic region (Kar et al., 2015; Chitimia-Dobler et al., 2017; Keskin, 2018; Bykov et al., 2023) or a specific ticks genus, but is rarely reported in Africa (Balinandi et al., 2019; Shuaib et al., 2020). Numerous studies have reported abnormalities in ixodid ticks such as *Amblyomma, Dermacentor, Haemphysalis, Hyalomma,* and *Rhipicephalus* (Okely et al., 2022). Within the *Ixodes* genus (Chong et al., 2020; Neumann, 1899), there are no data on the subgenus *Afrixodes* Morel, 1969 related to tropical Africa. In sum, of the 255 described species of the genus *Ixodes* (Guglielmone et al., 2023), only eight species have been found with morphological abnormalities (Tovornik, 1987; Alekseev and Dubinina, 1993; Larson and Paskewitz, 2016; Chitimia-Dobler et al., 2017; Chong et al., 2020; Bykov et al., 2023), and none of the species within *Afrixodes*, a specific subgenus found in Afrotropical area. This *Afrixodes*, including 67 species (Lorang and Augot, 2026), is a poorly known subgenus found mainly on wildlife, found in rainforest (Morel, 1969).

This study is the first report of morphological abnormalities in *Afrixodes* ticks collected in tropical Africa from wild mammals. This report highlights for the first time nymphal and female teratological specimens. Due to the lack of descriptions for 88% of species at the nymphal stage, 87% at the larval stage and 78% at the adult male stage, and the difficulty in morphologically identifying all species at the female stage, DNA barcoding was carried out on samples.

## 2 Material and methods

**Fig. 1:**
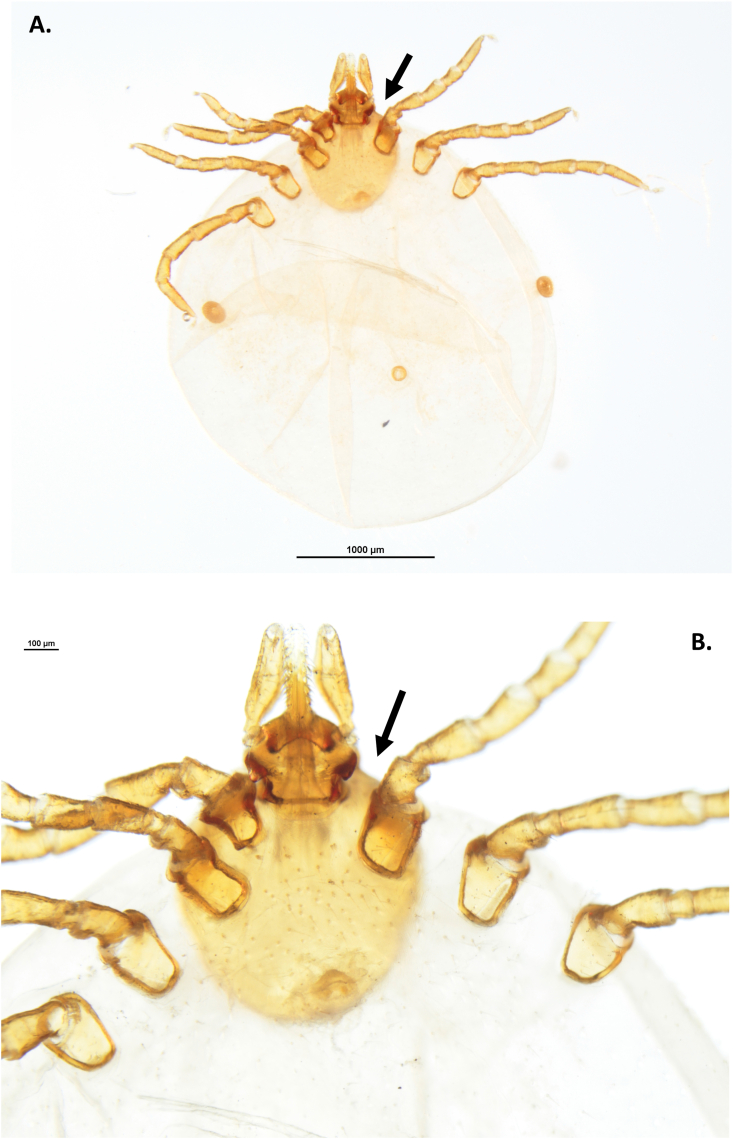
Female IF0132B, *Ixodes rasus*, ventral view. A. Global view (20X); B. Zoom on capitulum and coxae (60X). The arrow shows the ectromely (missing leg, first right).

**Fig. 2:**
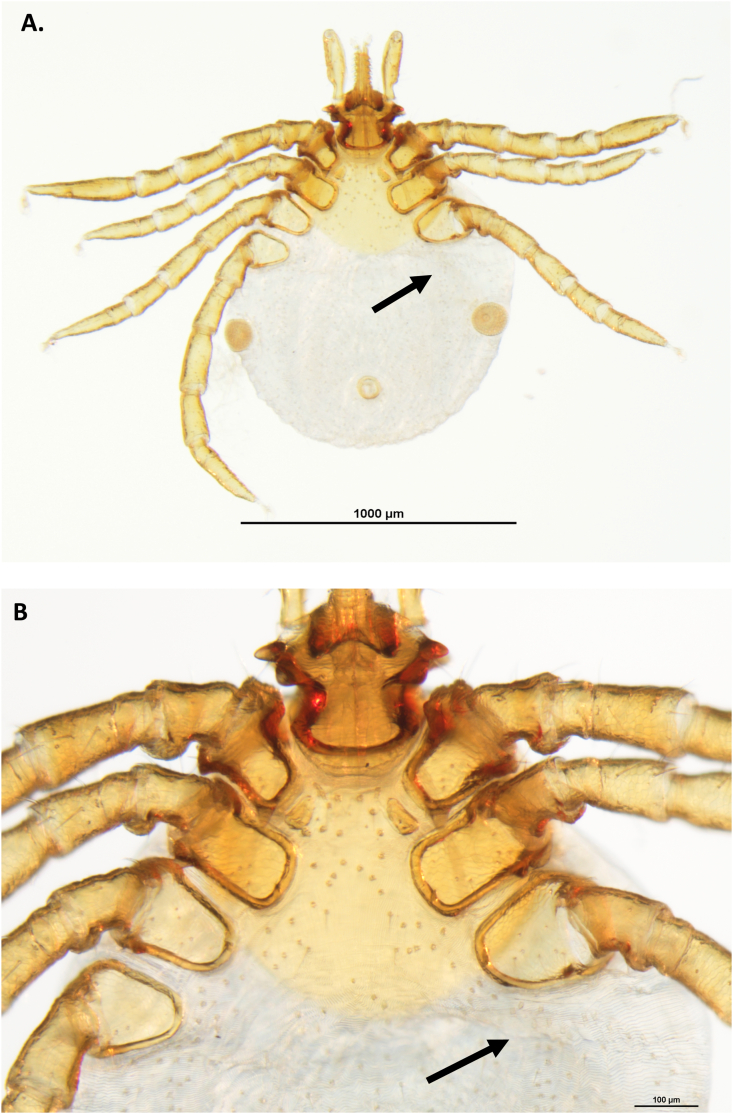
Nymph IN0057J, undescribed *Ixodes* (*Afrixodes*) sp., ventral view. A. Global view (30X); B. Zoom on capitulum and first coxae (110X). The arrow shows the ectromely (missing leg, last right).

**Fig. 3:**
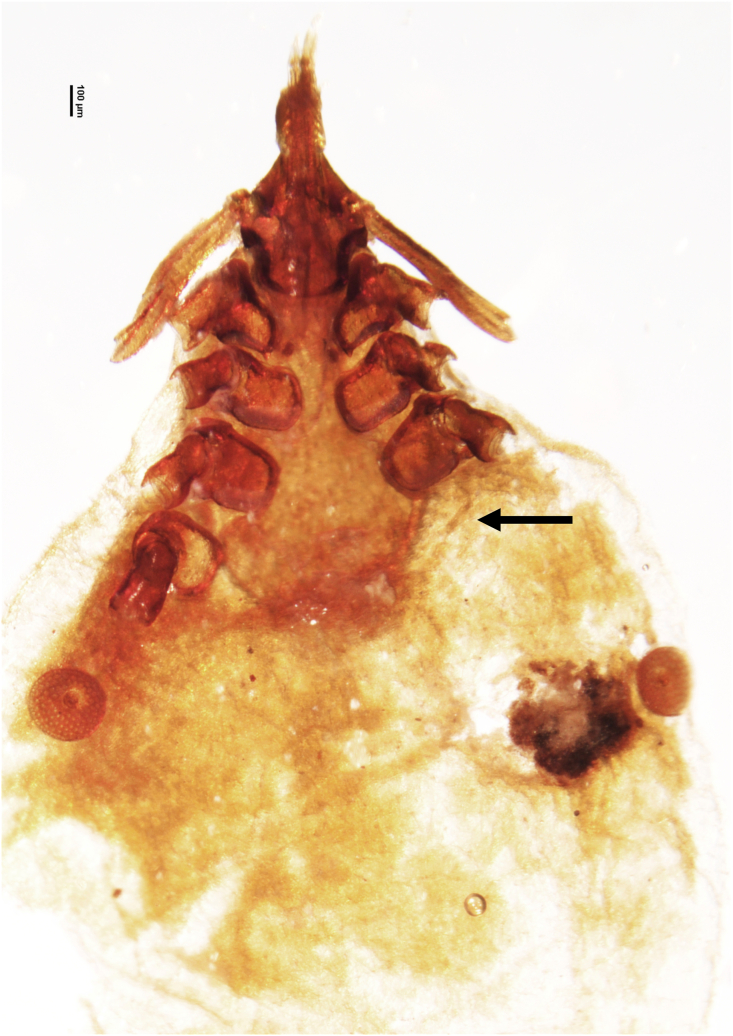
Nymph IN0603, undescribed *Ixodes* (*Afrixodes*) sp., ventral view. A. Global view (30X). The arrow shows the ectromely (missing leg, last right).

The ticks examined in this study were collected between 1998 and 1999 during different expeditions in the Central African Republic (CAR) and Gabon, focused on African wild animals. The female, IF0132B, was collected in Gabon in 1999 on a *Sylvisorex ollula* (Soricomorpha - Soricidae) (Fig. 1). Both nymphs were collected in CAR: IN0057J on *Hyemoschus aquaticus* (Artiodactyla - Tragulidae) in 1999 (Fig. 2) and IN0603 on *Hylomyscus* sp. (Rodentia - Muridae) in 1998 (Fig. 3). They originate from a larger collection of *Ixodes* (*A*.) ticks collected in seven countries of tropical Africa between 1995 and 2021 from 54 different animal hosts. These three ticks stand out among a collection of 82 females, six males, 413 nymphs and 171 larvae, i.e. 672 specimens. All the ticks were stored in 70% ethanol at +4°C. Specimens were identified through morphological analysis based on identification keys (Arthur, 1957, 1958, 1965; Arthur and Burrow, 1957; Clifford et al., 1975; Uilenberg et al., 1979; Spickett et al., 1981; Keirans et al., 1982; Matthysse and Colbo, 1987; Apanaskevich et al., 2011; Hornok et al., 2022). Photographs of these specimens were taken with a Nikon DS-Fi3 digital camera coupled to a Nikon SMZ25 stereomicroscope. For adult females, 20X and 60X magnifications were used, and for nymphs, 30X and 110X. To molecularly assign them to taxonomy status, 16S rRNA (Lorang et al., 2026) and cytochrome *c* oxydase subunit 1, Cox1 (Cruickshank, 2002) are used.

## 3 Results and discussion

**Fig. 4:**
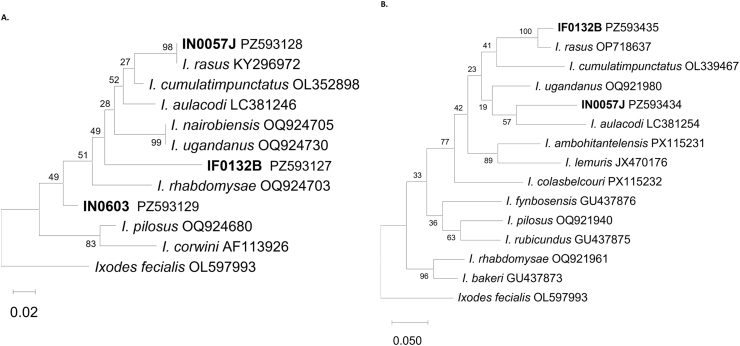
Maximum likelihood tree of 16S and Cox1 sequences for *Ixodes* (*Afrixodes*). The tree was inferred using Tamura model (1000 bootstrap replicates) and rooted with *Ixodes fecialis* (*Exopalpiger*) as an outgroup (MEGA 12.0.11). All others *Ixodes* species belonging to the *Afrixodes* subgenus. A. Phylogenetic tree of 16S gene; B. Phylogenetic tree of Cox1 gene.

Among the 677 *Ixodes* (*Afrixodes*) specimens from the global study, only one *I. rasus* female and two *Ixodes* (*A.*) spp. nymphs have a teratological abnormality. Fig. 4 (A and B) show the molecular analysis to the subgenus *Afrixodes* based on Cox1 and 16S RNA for the three specimens. The frequency of anomalies is 1.22% (1/82) of females and 0.5% (2/413*)* of nymphs. No male or larva was found with an anomaly. Thus, 0.45% of study specimens had a morphological abnormality. All of them have an ectromely and no other abnormalities were detected (Fig. 1, Fig. 2, Fig. 3). It is not excluded that sampling bias may be present in the collection of these ticks. Micromammals are hosts found in the ticks’ environment, enabling the ticks to complete their entire life cycle. In addition, the capture of a particular Mammalian species can favor particular stage or species of ticks. Nevertheless, these data allow us to better understand the biology of *Afrixodes*.This is the first report of abnormalities in *Afrixodes* ticks with a hight rate, regarding the current literature: i) Bykov et al. (2023) only found 0.001% (1/6892) of teratological abnormalities in their *Ixodes lividus* specimens, in a single larva; ii) Chitimia-Dobler et al. (2017) showed that 1.9% of specimens (*I. ricinus* and *I. inopinatus*), adults and nymphs, present an abnormalities, including ectromely; iii) Chong et al. (2020) found 0.01% of teratological nymphs, with *I. nipponensi*s presenting an ectromely; iv) Larson and Paskewitz, 2016 found 0.38% *I. scapularis* nymphs presented schizomely, asymmetry and ectromely; v) Alekseev and Dubinina (1993) found 2.48% *I. persulcatus* females present abnormalities with important deformations of the chitinous structure of scutum; vi) Alekseev et al. (2007) present 40% *I. persulcatus* with exoskeletal anomalies; vii) Kar et al. (2015) found 0.17% (33/18,667) of adults in *Hyalomma* spp., *Haemaphysalis* spp. and *Rhipicephalus* spp.; viii) Balinandi et al. (2019) found 0.004% in *Amblyomma* and *Rhipicephalus* adults, including ectromely; ix) and in the last example, but taking place also in Africa, is 0.62% for ticks from Sudan, with 13.4% of them (females) presenting an ectromely (Shuaib et al., 2020).

Abnormalities in ticks are likely underreported, and cases of teratology are more common than is reflected in the literature (Larson and Paskewitz, 2016). We encourage colleagues to publish these data in order to investigate the abiotic factors (geographical and environmental) that might explain these morphological changes. Experimentally, Buczek (2000) showed that when the temperature of tick eggs may cause abnormalities, including ectromely (15.4%, the third-highest rate of anomalies reported). Although the abnormalities observed in the post-molting stage are considered teratological, at least in the case of ectromely, it is not possible here to determine whether they are congenital in origin or whether they arise during the ticks' metamorphosis. One might think that these abnormalities could impair the tick's ability to feed on blood. However, these observations, this study and others (Keskin, 2018; Balinandi et al., 2019; Shuaib et al., 2020; Luz et al., 2023) show that ticks can parasitize animals despite their morphological abnormalities. They do not merely attach themselves to the animal, but actually feed (see engorged ticks on Fig. 1, Fig. 3).

As stated in the introduction, the aim here is also to show that such anomalies can be found on *Ixodes* parasitising wild animals. Other than the specimens studied here, Keskin (2018) found one *Ixodes frontalis* on a bird (*Turdus merula*) in Türkiye, such as Bykov et al. (2023) with one *I. lividus* larva in sand martin nest (*Riparia riparia*) in Iran. Luz et al. (2023) reports only *Amblyomma* on wildlife, with reports of one rodent, *Hydrochoerus hydrochaeris* (Caviidae), and one Artyodactyla but domestic one, *Bos taurus* (Bovidae). From domestic animals, reports are not concerning *Ixodes* and mostly collected from cattle: the *Rhipicephalus annulatus* founds by Keskin (2018); *Amblyomma lepidum* and *R. decoloratus* from Uganda by Balinandi et al. (2019); and various species of *Amblyomma*, *Hyalomma* and *Rhipicephalus* found by Shuaib et al. (2020).

In Africa, reports are located only in Sudan and Uganda but never deals with *Ixodes* or wildlife collections (Balinandi et al., 2019; Shuaib et al., 2020). These researches on the African continent could be the most geographically-related studies to ours, but here highlighting *Ixodes* (*Afrixodes*) ticks. Also, regarding host species, only Luz et al. (2023) in Brazil report a rodent and an Artiodactyla as hosts of teratological ticks. However, the rodent from which IN603 was collected belongs to the Muridae family, not the Caviidae as Luz et al. (2023) had suggested. Similarly, the bovine host infected with IF132B is a Tragulidae, not a Bovidae, as for Luz et al. (2023). Consequently, in addition to the first report on a shrew (IN0057J on Soricidae), these are also the first reports of malformed ticks found on Muridae and Tragulidae. Moreover, these are the first reports on these animals (shrew, rodent, and Tragulidae) in Africa as well.

## 4 Conclusion

Despite the difficulty working with the *Afrixodes* for their availability (collected from wildlife, mostly in rainforest) and their identification (lack of descriptions, and molecular data) this study reveals new insights about teratological abnormalities related to *Ixodes* (in Afrotropical area and collected on wildlife with new species of ticks, new host harbouring ticks, and new countries). Given the novelty of these findings, the *Afrixodes* subgenus warrants more in-depth studies of its species, particularly with regard to the description of stages (immature and male) and biomolecular research.

## Data statement

Scientific data will be made available on request.

## Funding

This work was supported by the 10.13039/501100003990Île-de-France region of France through the DIM OneHealth.

## CRediT authorship contribution statement

**Camille Lorang:** Data curation, Formal analysis, Investigation, Writing – original draft. **Denis Augot:** Formal analysis, Writing – review & editing.

## Declaration of competing interest

The authors declare that they no competing interest.
